# Adhesion of *Escherichia coli* and *Lactobacillus fermentum* to Films and Electrospun Fibrous Scaffolds from Composites of Poly(3-hydroxybutyrate) with Magnetic Nanoparticles in a Low-Frequency Magnetic Field

**DOI:** 10.3390/ijms25010208

**Published:** 2023-12-22

**Authors:** Vera V. Voinova, Vsevolod A. Zhuikov, Yulia V. Zhuikova, Anastasia A. Sorokina, Tatiana K. Makhina, Garina A. Bonartseva, Evgeniia Yu. Parshina, Muhammad Asif Hossain, Konstantin V. Shaitan, Artyom S. Pryadko, Roman V. Chernozem, Yulia R. Mukhortova, Lada E. Shlapakova, Roman A. Surmenev, Maria A. Surmeneva, Anton P. Bonartsev

**Affiliations:** 1 Faculty of Biology, Lomonosov Moscow State University, Moscow 119234, Russia; veravoinova@mail.ru (V.V.V.); anastasiasorokina251@gmail.com (A.A.S.); parshinae@gmail.com (E.Y.P.); asifhossain38@yahoo.com (M.A.H.); shaytan49@yandex.ru (K.V.S.); 2 The Federal Research Centre “Fundamentals of Biotechnology” of the Russian Academy of Sciences, Moscow 119071, Russia; vsevolod1905@yandex.ru (V.A.Z.); zhuikova.uv@gmail.com (Y.V.Z.); tat.makhina@gmail.com (T.K.M.); bonar@inbi.ras.ru (G.A.B.); 3 Physical Materials Science and Composite Materials Center, Research School of Chemistry and Applied Biomedical Sciences, National Research Tomsk Polytechnic University, Tomsk 634050, Russia; vilajer@gmail.com (A.S.P.); phenics100@gmail.com (Y.R.M.); les2@tpu.ru (L.E.S.); rsurmenev@mail.ru (R.A.S.); surmenevamaria@tpu.ru (M.A.S.); 4 International Research and Development Center “Piezo- and Magnetoelectric Materials”, Research School of Chemistry and Applied Biomedical Sciences, National Research Tomsk Polytechnic University, Tomsk 634050, Russia; rvc1@tpu.ru

**Keywords:** poly(3-hydroxybutyrate), magnetic nanoparticles, graphene oxide, scaffolds, *E. coli*, *L. fermentum*, adhesion, low-frequency magnetic field, electroactive biomaterial, piezoelectricity

## Abstract

The ability of materials to adhere bacteria on their surface is one of the most important aspects of their development and application in bioengineering. In this work, the effect of the properties of films and electrospun scaffolds made of composite materials based on biosynthetic poly(3-hydroxybutyrate) (PHB) with the addition of magnetite nanoparticles (MNP) and their complex with graphene oxide (MNP/GO) on the adhesion of *E. coli* and *L. fermentum* under the influence of a low-frequency magnetic field and without it was investigated. The physicochemical properties (crystallinity; surface hydrophilicity) of the materials were investigated by X-ray structural analysis, differential scanning calorimetry and “drop deposition” methods, and their surface topography was studied by scanning electron and atomic force microscopy. Crystal violet staining made it possible to reveal differences in the surface charge value and to study the adhesion of bacteria to it. It was shown that the differences in physicochemical properties of materials and the manifestation of magnetoactive properties of materials have a multidirectional effect on the adhesion of model microorganisms. Compared to pure PHB, the adhesion of *E. coli* to PHB-MNP/GO, and for *L. fermentum* to both composite materials, was higher. In the magnetic field, the adhesion of *E. coli* increased markedly compared to PHB-MNP/GO, whereas the effect on the adhesion of *L. fermentum* was reversed and was only evident in samples with PHB-MNP. Thus, the resultant factors enhancing and impairing the substrate binding of Gram-negative *E. coli* and Gram-positive *L. fermentum* turned out to be multidirectional, as they probably have different sensitivity to them. The results obtained will allow for the development of materials with externally controlled adhesion of bacteria to them for biotechnology and medicine.

## 1. Introduction

The development of biomimetic materials for medical applications is a promising trend in bioengineering. In particular, considerable attention has been paid to the use of electroactive biomaterials that mimic the electrophysiological microenvironment characteristic of following tissues, bone, cartilage, tendon, skin, ligament, and sclera, that have inherent piezoelectricity [1]. It is connected with the electromechanical coupling of collagen in these tissues, whose α-helices are regular structures of anisotropic crystals [1,2]. In this regard, biomaterials that exhibit piezoelectricity are being developed for regenerative medicine of bone and other tissues [3,4,5,6,7]. Electroactive materials used to mimic the extracellular matrix of electroactive tissues should also possess piezoelectric properties or electrical conductivity [7,8,9,10]. In this work, our attention is focused on an electroactive polymeric biomaterial produced by bacterial biosynthesis, poly(3-hydroxybutyrate) (PHB). This polymer has been shown to exhibit piezoelectricity, with the piezoelectric constant (of pure PHB) being comparable to that of collagen: d_14_ is 1.6–2.0 pC/N [4,9]. It gives PHB and PHB-based biomaterials’ biomimetic properties. Due to the combination of physicochemical properties, biocompatibility and biodegradability [11,12,13], PHB is a promising material for tissue engineering.

Piezoelectric properties of anisotropic dielectrics, which include PHB, manifest themselves in the form of charge generation in response to mechanical deformation. In experiments aimed at studying piezoelectric effect (PEE), the challenge arises to realize such electromechanical coupling. One of the approaches that allows us to do this is to modify the biomaterial composition by adding a magnetically active component, most often magnetite (Fe_3_O_4_) nanoparticles (MNP), followed by an alternating magnetic field (MF) [14]. In this system, the mechanical stress from the ferromagnetic nanoparticles under the force effect of an external alternating MF characterized by a variable magnetic induction vector is transferred to the polymer material, thus causing the so-called direct PEE. We sought to realize this approach in our work. In addition, we added partially reduced graphene oxide (GO) into the composite material [15,16]. In such a complex, the GO sheets appear to be more conductive than the polymer, which leads to charge mobilization around the perimeter of the particles, leading to heteropolarization in the material, which enhances the observed electrical effect [17].

However, it is highly difficult to directly measure the piezo effect, or at least to confirm it with the help of some additional equipment, under conditions that electromechanical coupling is realized when samples of polymeric materials are exposed to an alternating magnetic field inside a magnetic device. Since it is known that the piezo effect can influence the binding of bacteria to the surface of materials [18], we responded to this challenge by developing a model in which the degree of bacterial adhesion serves as an indicator of the manifestation of the piezo effect. This work allowed us to compare the piezoelectric properties of one- and two-component composite materials based on PHB under the influence of a low-frequency magnetic field (LFMF).

One of the most important aspects of the development and application of biomaterials in regenerative medicine is the nature of their interaction with bacteria. This determines the increasing interest in studying the interaction of bacteria with electroactive materials. Stimulation of PEE is considered as an innovative approach to tailoring bacteria response and creating antimicrobial defense [18]. Currently, there is no model that well describes the adhesion of all species and strains of bacteria to different surfaces. Accordingly, it would seem very difficult to predict accurately enough how adhesion will be affected by material modification, since it is an integrated process reflecting the contribution of many factors, such as micro and nanotopography, hydrophobicity, charge, etc., as well as the properties of the microorganisms themselves. Even minor changes in the composition of the PHB-based material, such as varying the molecular weight of the polymer, obtaining copolymers and composites based on it, and even the technology of polymer device manufacturing are reflected in the physicochemical properties of the biomaterial and the piezoelectric response [19]. At the same time, each of the physicochemical factors may have different importance for different microorganisms which determines their selective effects. As a result, information regarding the contribution of each factor individually can be contradictory [20,21]. The influence of the electrical properties of PHB on bacterial adhesion has not yet been studied. The lack of knowledge in this area motivates further research in this direction, since understanding how the physicochemical properties of the material affect the initial phase of bacterial adhesion is essential for the development of bioengineered materials with improved antibacterial properties and can serve as a basis for optimizing the surface properties of the material according to a specific application. To address these challenges, we investigated how the physicochemical properties of films and electrospun scaffolds made of pure PHB and composite materials based on it affect the adhesion of bacteria to their surface under the influence of a LFMF and without it.

## 2. Results and Discussion

### 2.1. Effect of Low-Frequency Magnetic Field on the Density of Bacterial Cultures

**Figure 1:**
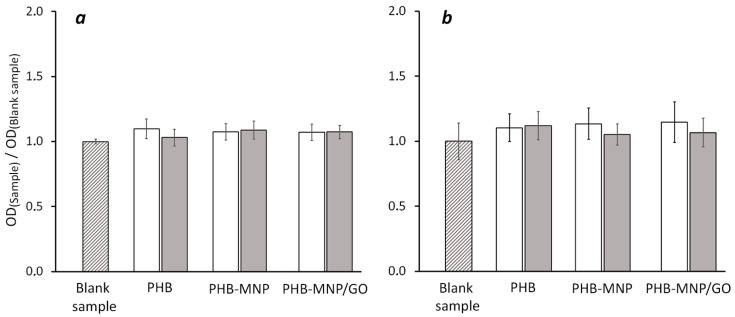
Relative value of OD at 600 nm of suspension cultures of *L. fermentum* (**a**) and *E. coli* (**b**) in samples not exposed to LFMF without polymeric material (shaded bars), not exposed to LFMF with polymeric material (white bars), and exposed to LFMF (*B_m_* = 68 mT, *f* = 0.67 Hz) with polymeric material (gray bars). The samples are films from pure PHB, composite PHB-MNP and complex PHB-MNP/GO. The OD_(blank sample)_ for *L. fermentum* was 7.4 and for *E. coli,* it was 1.08. The results of eight independent experiments are presented.

We investigated the effect of a LFMF (*B_m_* = 68 mT, *f* = 0.67 Hz) on the density of suspension cultures of *E. coli* and *L. fermentum* cultured together with films and scaffolds made of pure PHB and its composites with magnetite PHB-MNP and the complex of magnetite nanoparticles with partially reduced graphene oxide PHB-MNP/GO. The growth of bacterial cultures was accompanied by a uniform turbidity of media, which is characteristic of facultative anaerobes, while the optical density (OD) of bacterial suspensions indirectly reflected the number of cells in them (Figure 1).

The density of the *L. fermentum* suspension culture (Figure 1a) after 16 h of incubation with polymer films, both without and with the influence of the LFMF, was not statistically different from the density of the culture in control samples containing no samples and not exposed to the MF. Similar results were obtained for the *E. coli* culture (Figure 1b), which was incubated for 24 h.

Free nanoparticles of magnetite, graphene and its derivatives are able to exert a bactericidal effect [22,23]. However, in our experiments, in the absence of the LFMF, the samples made of pure PHB, as well as composite films, did not cause an effect on the growth of the bacterial cultures. Apparently, the bactericidal effect occurs in the case of direct contact between nanoparticles and bacterial membranes, whereas in our experiment, the polymeric material shielded the nanoparticles and retained them in its matrix, preventing their diffusion into the culture medium and thus reducing their toxicity.

Microorganisms can be sensitive to MFs. The analysis of results in this field of magnetobiology is complicated by the fact that, to date, there is no “gold standard” of conditions for conducting experiments to study the effect of a magnetic field on bacterial growth and adhesion. We compared our results with those previous works in which the values of magnetic induction and magnetic field frequency were close or comparable to ours. The research works devoted to the study of the proliferation and viability of bacteria under the influence of a low-frequency (˂300 Hz) or constant MF with moderate magnetic induction (from 1 mT to 1 T) showed contradictory results. Thus, in a number of investigations, a bactericidal effect was revealed. In the study of Ji et al., 30 min exposure to a constant MF with induction of 45 and 450 mT reduced the number of colony-forming units (CFU) in the suspension culture of *E. coli* by 40–60% (the effect was more pronounced at a higher value of magnetic induction) [24]. Similar results were obtained at 60–120 min of exposure to an alternating MF (*B_m_* = 10 mT, *f* = 50 Hz): the number of CFU in the suspension culture of *E. coli* decreased by 30–50% [25,26]. There are also studies in which it was shown that exposure to a constant MF (*B_m_* = 95 mT, *t* = 3 h) [27], (*B_m_* = 159 mT, *t* = 24 h) [28] has no effect on *E. coli* culture density, or that the effect of exposure to a rotating MF (*B_m_* = 30 mT, *f* = 50 Hz) is pronounced only in the phase of rapid bacterial growth and is leveled out at later stages [29], which correlates with our results. There is also evidence that a LFMF with a magnetic induction value of 25 to 55 mT and a frequency of approximately 16 mHz [30] and 50 Hz [29,31] can have a stimulating effect on bacterial growth, increasing by 25 to 40% the culture density and CFU values of bacteria, including *E. coli*. The exposure time in these studies varied from 1 to 12 h.

The effect of the MF on the density of the suspension culture and the CFU value of bacteria depends on the frequency, induction value and exposure time of the MF, as well as on microorganism strain. Under the same conditions for different microorganisms, the effect may be expressed to different degrees or even absent [25,31]. Some bacterial species, including *E. coli*, are more sensitive to the MF than others. In a number of studies, it was shown that this dependence can have points of extremum both on the time scale [29,30] and on the scale of magnetic induction values [24,32]. In Tokalov and Gutzeit, this phenomenon is called the “window effect” [33].

### 2.2. Study of Bacterial Adhesion

The pattern of bacterial adhesion to films and scaffolds was uniform for each of the microorganisms studied and differed only in the severity of the effects.

For each species of model microorganisms, the OD value of crystal violet extracts, after staining polymeric samples (films or scaffolds of pure PHB) that were not exposed to a LFMF, was denoted by OD_(Control)_. The corresponding numerical value for *L. fermentum* on films was 2.82; for *L. fermentum* on scaffolds, it was 9.88; for *E. coli* on films, it was 0.16; for *E. coli* on scaffolds, it was 0.83. The OD values of all other samples were denoted by OD_(Sample)_.

**Figure 2:**
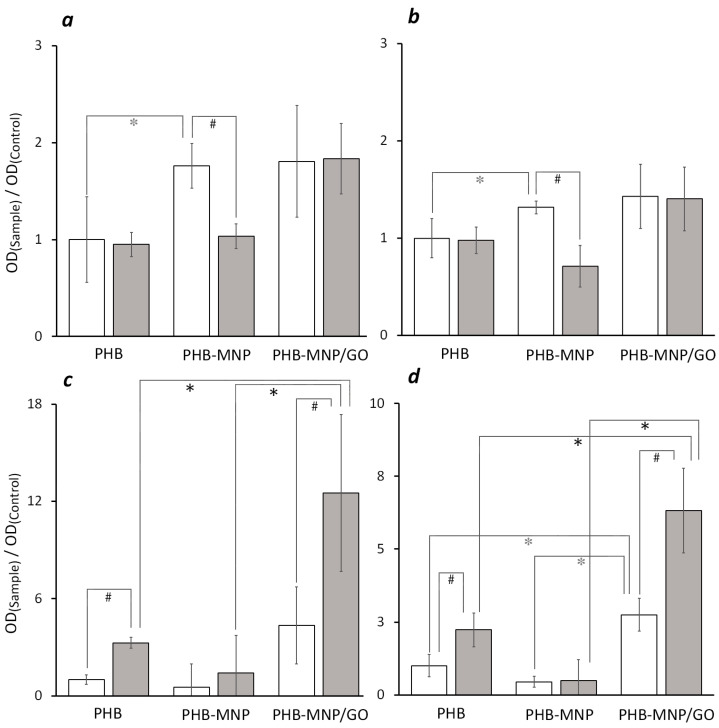
Relative value of OD (λ = 565 nm) of crystal violet extracts. Samples: films (**left** (**a**,**c**)) and scaffolds (**right** (**b**,**d**)) were incubated with *L. fermentum* (**top** (**a**,**b**)) and with *E. coli* (**bottom** (**c**,**d**)). Bacteria were cultured without MF (white bars) and under the influence of LFMF (*B_m_* = 68 mT, *f* = 0.67 Hz) (gray bars). The OD_(Control)_ value for *L. fermentum* on films was 2.82; for *L. fermentum* on scaffolds, 9.88; for *E. coli* on films, 0.16; for *E. coli* on scaffolds, 0.83. * *p* ˂ 0.05 PHB-MNP/GO vs. PHB, and PHB-MNP vs. PHB (for –MF); * *p* ˂ 0.05 PHB-MNP/GO vs. PHB, and PHB-MNP/GO vs. PHB-MNP (for +MF); ^#^ *p* ˂ 0.05 +MF vs. –MF (for PHB, PHB-MNP and PHB-MNP/GO); *n* = 8.

Comparison of the OD_(Control)_ values, which reflect the bacterial adhesion properties of films (Figure 2a,c) and scaffolds (Figure 2b,d) from PHB, showed that three to five times more bacterial cells adhere to the surface of the scaffolds than to the films on both bacterial models. This difference is predictable because the scaffolds are 3D structures built by multiple interwoven filaments, providing a larger area for bacterial colonization compared to the surface of plane films of the same size.

The addition of magnetic nanoparticles to the polymeric biomaterial had different effects on the adhesion of model microorganisms. The number of *L. fermentum* that bound to films and scaffolds from both composites in the absence of the LFMF was greater compared to PHB (the difference was not statistically significant in PHB-MNP/GO samples) (Figure 2a,b, white bars). The percentage of *E. coli* that bound to PHB-MNP films and scaffolds did not differ from the control, but when MNP were added in complex with GO, the adhesion level increased (Figure 2c,d, white bars).

Exposure to the LFMF did not affect the adhesion of *L. fermentum* to pure PHB (not containing magnetically active nanoparticles) films and scaffolds but resulted in an increase in *E. coli* adhesion. The effect of the LFMF on the adhesion of *E. coli* to pure PHB samples was apparently not mediated by a change in the properties of the polymeric material; in the absence of magnetically active nanoparticles, exposure to a LFMF did not cause a PEE on the surface of the polymeric samples. It was probably due to the direct effect of the LFMF on bacterial cells. As mentioned above, a MF, depending on the time of exposure, magnitude of magnetic induction and frequency, can influence the kinetics of bacterial growth.

In addition, there is evidence in the literature that a MF can also affect bacterial adhesion and biofilm formation [34]. As in the case of the effect of a MF on bacterial growth kinetics, the effect of a MF on bacterial adhesion can be multidirectional. Thus, Fijałkowski et al. showed that the effect of a rotating MF (*B_m_* = 25–34 mT, *f* = 5–50 Hz) stimulated the binding of *E. coli* to the surface of samples, which is consistent with our data, but at the same time prevented the adhesion of Gram-negative *Acinetobacter baumannii* and *Pseudomonas aeruginosa* [31]. In the work of Chua and Yeo, a constant MF had both stimulating and suppressive effects on the biofilm formation of Gram-positive *Bacillus licheniformis* depending on the direction of magnetic induction lines relative to the substratum [35]. According to Di Campli et al., a low-frequency MF suppressed biofilm formation by Gram-negative *Helicobacter pylori* (*B_m_* = 1 mT, *f* = 50 Hz) [36]. Currently, there are several theoretical concepts of magnetobiology that explain such non-specific biological effects of a MF on living systems through the influence on the molecular mechanisms of chemical processes [37].

Analyzing the results presented in Figure 2, it is important to note that the observed effects modulating the bacterial adhesion level reflect the additive influence of three components of the experimental system: the physical and chemical properties of the surface of polymeric films and scaffolds, the LFMF effect and the polarization of the surface of polymeric samples due to the PEE. Since for *L. fermentum,* we have shown that the MF did not affect adhesion, this factor can be disregarded in this bacterial model. The binding of bacteria to the composite films and scaffolds did not differ in the absence of the MF. Consequently, the decrease in the adhesion of *L. fermentum* to the PHB-MNP composite in the LFMF was probably due to the manifestation of the anti-adhesive effect of the PEE. The lack of difference in the level of adhesion of *L. fermentum* to the PHB-MNP/GO films and scaffolds with and without the LFMF suggests that the PEE was much weaker on this material (Figure 2a,b).

On the one hand, in the samples with *E. coli,* the LFMF enhanced the binding of bacteria to PHB samples, which did not show any PEE. On the other hand, the addition of MNP to the composite material did not affect bacterial adhesion. Hence, the manifestation of PEE in the presence of the LFMF in the PHB-MNP samples prevented additional binding of bacteria to their surface (Figure 2c,d). The surface properties of the PHB-MNP/GO composite material were responsible for the higher adhesion of *E. coli* compared to the pure PHB material. Together with the adhesion-enhancing effect of the MF, the additive effect of these two factors had a greater influence on *E. coli* binding than the anti-adhesive effect of the PEE in this sample, resulting in an increase in the number of bound bacteria in the presence of the LFMF. The obtained results are in good agreement with the literature data. Carvalho et al. showed that the PEE on the substrate surface did not affect the adhesion of Gram-negative *E. coli* but had a bacteriotoxic effect on Gram-positive *Staphylococcus epidermidis* [38]. Another work showed the antibacterial effect of piezoelectric polarization on *E. coli* and *Staphylococcus aureus* [39]. Vatlin et al. demonstrated the bacteriostatic effect exerted by piezoelectric polymeric materials, PHB and Polyvinylidene Fluoride, exposed to ultrasound on *E. coli* [40].

### 2.3. Physicochemical Properties of Materials

The question of what factors influence the process of surface colonization of various materials by bacteria has been widely investigated. It is believed that the primary attachment of microorganisms is determined by the conditions of the microenvironment, the combination of physicochemical properties of the material and physicochemical properties of the bacterial cell wall, and can be considered as the adhesion of microparticles to solid surfaces based on the theory of colloidal chemistry [41,42].

#### 2.3.1. Surface Topography

**Figure 3:**
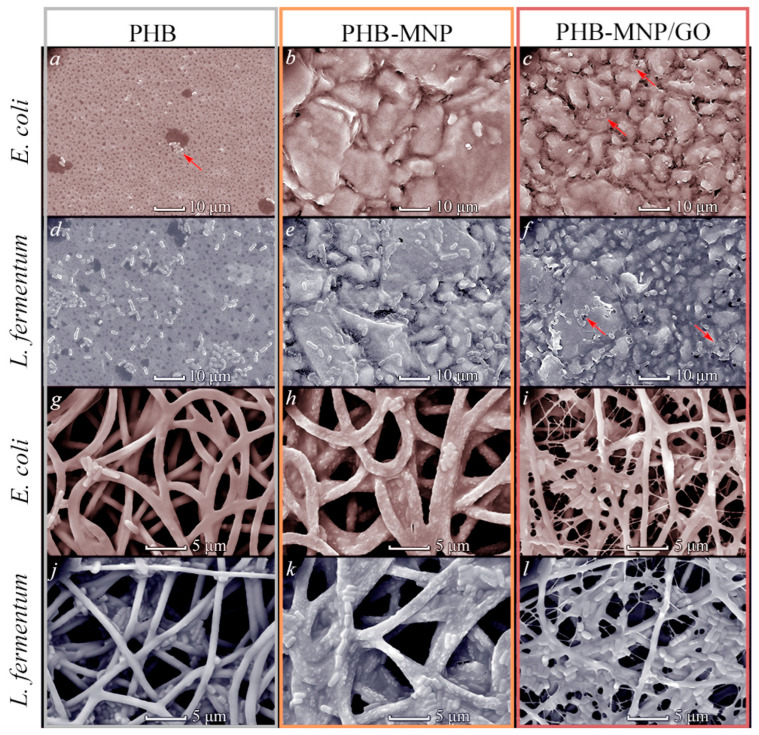
Adhesion of *E. coli* (red hue, (**a**–**c**,**g**–**i**)) and *L. fermentum* (blue hue, (**d**–**f**,**j**–**l**)) onto films and scaffolds of PHB ((**a**,**d**,**g**,**j**), respectively), PHB-MNP ((**b**,**e**,**h**,**k**), respectively) and PHB-MNP/GO ((**c**,**f**,**i**,**l**), respectively). Red arrows indicate the locations of bacterial cell clusters.

Surface topography (microtopography) and roughness (nanotopography) are important factors affecting bacterial adhesion [43]. We examined the surface microtopography of our samples using scanning electron microscopy (SEM). Figure 3 shows images of polymer samples incubated with bacteria without magnetic field exposure. The PHB films had a relatively smooth surface, with many small depressions with a diameter of about 0.8 μm and a few larger depressions with a diameter of 5–10 μm in the field of view. The nanoparticle composites were noticeably more uneven. At the same time, the PHB-MNP/GO film was characterized by the greatest lumpiness.

The presence of the surface folds is an important condition for the successful attachment of bacteria. They increase the contact area of the substratum with bacterial cells and provide them with shelter from external factors, such as hydrodynamic flows that can wash the cells off the surface [44]. Smooth surfaces (with roughness index *R_a_* of 2 μm and below) of bacteria bind worse than rougher surfaces [42].

Indeed, Figure 3a shows that *E. coli* cells, which colonized the pure PHB films significantly less actively than *L. fermentum*, localized mainly in the recesses (Figure 3a). In this respect, the structure of the scaffolds appears to be more favorable for bacterial multiplication than the surface of the films. The intertwined filaments and their contact points create partially isolated internal compartments. However, it can be seen (Figure 3g–l) that bacteria inhabited mainly the surface layers of the scaffolds, probably due to the fact that both strains are not mobile.

Surface structure at the submicron scale can also influence bacterial adhesion. Bumpy and corrugated surfaces with height differences of tenths of microns are characterized by a reduced area of contact with bacterial cells, which is also a factor preventing bacterial adhesion. The presence of sharp protrusions on the surface with linear dimensions of the vertices being hundredths of microns and sufficiently far apart from each other may give them bactericidal properties, presumably due to the fact that bacterial membranes are stretched and damaged upon contact with them. This is more important for Gram-positive bacteria than for Gram-negative bacteria due to differences in their cell wall structure [45,46,47]. In some studies, the bacteriotoxicity of graphene nanoplates possessing sharp edges is also attributed to this effect [23,48]. This mechanism has been termed “sharp edge mediated insertion”. However, for the manifestation of such a bactericidal effect, apparently, the mutual location of the “sharp” structures and the distance between them are important. Thus, Zhang et al. showed that the surface densely covered with vertically oriented graphene plates did not have a bactericidal effect on *E. coli* and Gram-positive *S. aureus* [49].

**Figure 4:**
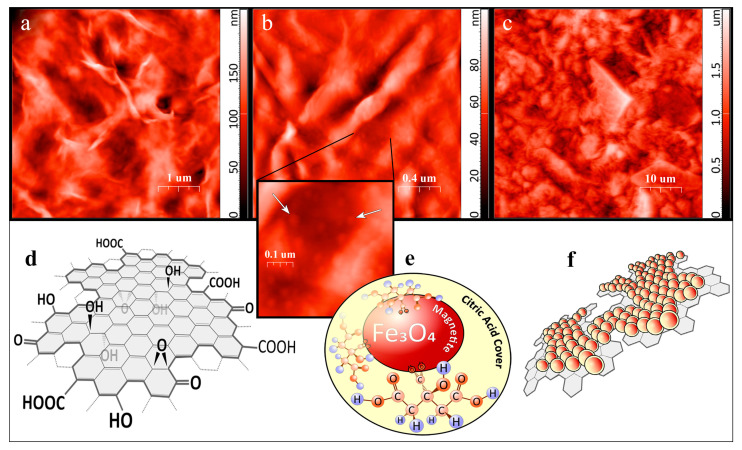
Topography of the upper (**a**,**b**) and lower (**c**) surface of polymer films: (**a**) PHB; (**b**) PHB-MNP; (**c**) PHB-MNP/GO, analyzed by AFM. Schematic representation of structural elements of composite materials: (**d**) partially reduced graphene oxide (GO); (**e**) covalent bond between magnetite nanoparticle and citrate; (**f**) magnetite nanoparticles in citrate shells attached to the surface of plate of partially reduced graphene oxide. In the enlarged image inset to (**b**), bumps with a size comparable to that of magnetite nanoparticles are indicated by white arrows.

Complex MNP/GO nanoparticles in the PHB-MNP/GO material, the structural elements of which are schematically depicted in Figure 4d–f, were partially reduced graphene oxide nanoplates 800 ± 500 nm in size, on the surface of which rounded Fe_3_O_4_ nanoparticles in citrate shells with a diameter of 22 ± 5 nm were uniformly distributed (according to SEM, Figure S2c).

Based on the concept of “sharp edge mediated insertion” of bacterial cells [23,48], it can be assumed that the sharp edges of graphene nanoplatelets extending above the surface of the material can have a bactericidal effect. We investigated the nanotopography of the surface of polymer films by atomic force microscopy (AFM) (Figure 4a–c). Films made of pure PHB were the least rough. The addition of magnetite nanoparticles to the composite material resulted in a slight increase in the maximum peak height on the AFM scan. On their surface, bumps with a size comparable to that of magnetite nanoparticles were distinguishable (indicated by white arrows in Figure 4b). The surface of PHB-MNP/GO films was characterized by the largest height differences due to the formation of folds. Their AFM scans (Figure 4c) showed structures morphologically similar to the composite of partially reduced graphene oxide and magnetite nanoplates. However, their density on the surface was negligible. This indicates that the MNP/GO complex structures were located predominantly in the thickness of the polymer material.

It is important to note that during the fabrication of films through the precipitation of polymers from the solution onto the glass surface, their upper side dries in contact with air, while the lower side dries adjacent to the glass. Related to this is the difference in the roughness of these two sides, which was previously demonstrated by us [50,51] and has been confirmed in the literature [52]. The roughness of the top side of the film depends on the intensity with which solvent evaporation from its surface took place. Examples of roughness profiles of polymer films are shown in Figure S3a–c. The roughness coefficients of the profile of the film surfaces (*R_a_*) are summarized in Table 1. According to these data, the roughness of the top side of PHB-MNP/GO films is sufficiently pronounced to prevent bacterial adhesion to its surface by reducing the contact area with bacterial cells.

The micrometer-scale irregularities and folds on the surface of the composite materials and their roughness may be the reason why bacteria bound to them better than to pure PHB. This was valid for *L. fermentum* adhesion to both composite samples and for *E. coli* adhesion to the PHB-MNP/GO sample (Figure 2). However, the adhesion of *E. coli* to the PHB-MNP composite did not differ from the control. This result reflects the circumstance that surface topography is only one of the factors, such as a change in surface charge, affecting bacterial adhesion. For *E. coli* adhesion to the PHB-MNP composite against other factors, this one was probably insignificant.

#### 2.3.2. Physicochemical Properties of Materials

Initial bacterial adhesion is largely determined by the combined effect of hydrophobic and electrostatic cell–substrate interactions [53,54]. We analyzed whether the addition of MNP and MNP/GO to the polymer material composition affects these substratum properties. The results of surface hydrophilicity properties (*θ*) of the polymer film samples and the model bacterial strains that we used are presented in Table 2. Water contact angle profiles of the upper side of polymer films are presented in Figure S3d–f.

According to Vogler, materials whose surface water contact angle value exceeds 65° are considered hydrophobic [55]. Table 2 shows that all analyzed films exhibited hydrophobic properties, whereas the surface of both bacterial strains was hydrophilic. The *θ* values of pure PHB and PHB-MNP composite films did not differ, while the PHB-MNP/GO sample had the higher *θ* value with statistical significance (*p* ˂ 0.05). Moreover, all samples had higher contact angle values on the top side of the films than on the bottom side.

It is known that the contact angle value does not directly determine the hydrophobicity of a material, since its value is affected by the physical properties of the surface: its roughness, degree of crystallinity and chemical heterogeneity [56]. Above, we have already mentioned the difference in roughness of the studied polymer films. The chemical heterogeneity factor can also have an effect on the *θ* value of composite materials, since their constituents differ significantly in the free surface energy value. In addition, the supplementation of nanoparticles in the polymer material could affect its crystallinity. The crystalline components in the PHB composition have a higher molecular density and hence a differing free surface energy value than the amorphous components.

Table 2 shows the *Sdr* values of pure PHB and composite films determined by AFM, as well as the θ* values found from relation (4). Considering the differences in the roughness of the films, the values of the contact angle of the composite materials were lower than the pure PHB, which was expected based on the formulation of the composites [15,57]. It can also be seen that the θ* values of the contact angles of the top and bottom sides of the films for each of the samples are equalized.

The influence of substrate hydrophobicity on bacterial adhesion was demonstrated in Oh et al. When comparing the level of adhesion of *E. coli* and *S. aureus* to surfaces characterized by low roughness and differing in hydrophobicity and charge, the authors showed that the adhesion level of both strains was maximal to hydrophobic materials, for which the contact angle took values around 100°. With decreasing hydrophobicity and increasing negative surface charge, the level of bacterial adhesion decreased [58]. Similar results confirming that hydrophilic materials are more resistant to bacterial adhesion than hydrophobic materials have been demonstrated in other earlier research [59,60]. In our experiments, the values of the contact angles that we found by considering the surface roughness of the PHB-MNP and PHB-MNP/GO composite films differed by less than two degrees from the value for the pure PHB film. This slight change in the value of θ* should not have affected the level of bacterial adhesion.

**Figure 5:**
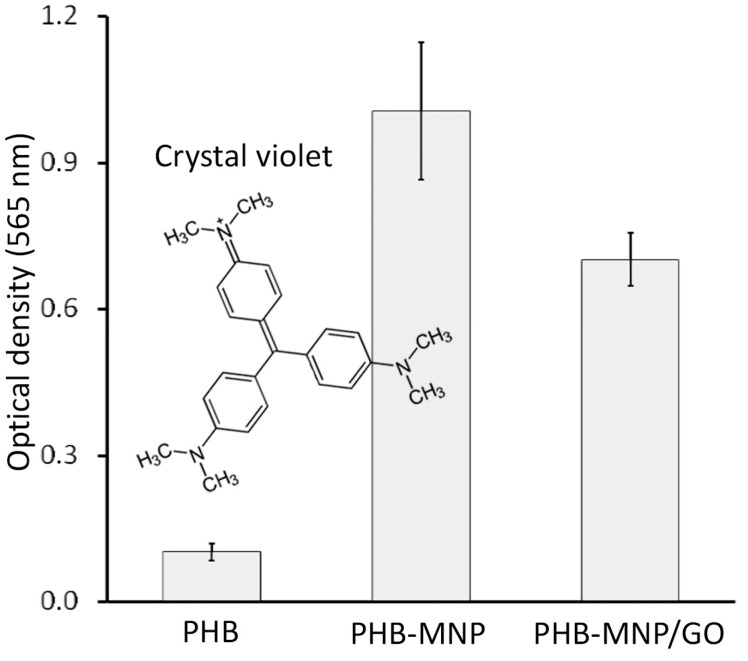
Sorption of crystal violet to the surface of films made of pure PHB and composites PHB-MNP and PHB-MNP/GO. The differences of the values are statistically reliable (*p* ˂ 0.05).

It could be expected that exposure of ionizable carboxyl groups of citric acid, a component of MNP, and other oxygen-containing groups to the surface of the polymeric material could lead to the appearance of a negative charge on it. The fairness of this assumption is confirmed by the fact that crystal violet, being the basic dye, bound to a greater extent to composite materials than to films made of pure PHB (Figure 5). At the same time, the adhesion level of crystal violet to the PHB-MNP sample was higher than to the PHB-MNP/GO sample, probably due to the fact that MNP associated with graphene oxide nanoplatelets were exposed to the surface of the polymer material to a lesser extent.

The more hydrophilic the bacteria and the substrate, the more important the electrostatic interactions between them [61,62]. Most bacterial cells are negatively charged. Bacteria belonging to different strains of the same species can differ significantly in the magnitude of surface charge, which determines the sensitivity of their adhesion in response to changes in surface charge [63]. Wilhelm et al. compared the negative charge density of *E. coli* mc4100 and *L. ramnosus* R0011 and showed that in the first bacterial species, its density is seven times higher than in the second [64]. The presence of homonymous (negative) charge on the substrate surface leads to electrostatic repulsion of microorganisms [60].

In our experiments, the appearance of negative charge on the surface of composite polymeric materials apparently inhibited the adhesion of *E. coli* to their surface; the effect was more pronounced when bacteria interacted with the PHB-MNP material, whereas *L. fermentum* was insensitive to this factor.

#### 2.3.3. Crystallinity and Piezoelectric Effect

The degree of crystallinity of polymer films and scaffolds was quantitatively evaluated using differential scanning calorimetry (DSC) (Table 3, Figure S1). It should be noted that the differences in the manufacturing technologies of films and scaffolds cause differences in their thermal properties. The crystallinity of films is higher than that of scaffolds made from the same material. A similar conclusion can be based on the shift of the 1719 band (C=O stretching in the crystalline phase) in the scaffolds to the region of higher frequencies on FTIR spectra (Figure S4) [65,66,67]. The addition of nanoparticles to the PHB-based composite polymer materials had practically no effect on their melting temperature but resulted in a decrease in crystallinity by 14–19%, as it follows from the data of thermal analysis.

**Figure 6:**
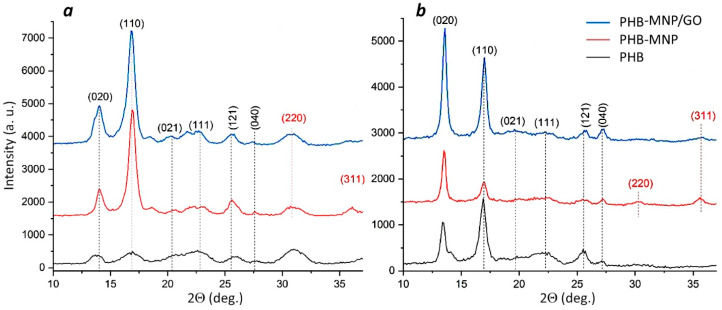
XRD patterns of films (**a**) and scaffolds (**b**) made of PHB, PHB-MNP and PHB-MNP/GO.

The crystalline structure of the polymer samples was investigated by X-ray diffraction (XRD) analysis (Figure 6). The XRD spectra of each of the studied samples show reflections characteristic of the orthorhombic α-phase of PHB, namely, peaks at angles 2*Θ* 13.6°, 16.9°, 19.9°, 22.4°, 25.5° and 26.9° of the crystallographic planes (020), (110), (021), (111), (121) and (040), respectively. In the case of the PHB-MNP and PHB-MNP/GO composite films and scaffolds, magnetite reflections with a spinel face-centered lattice are also observed at 30.35° and 35.63°, corresponding to planes (220) and (311). At the same time, no reflexes corresponding to OG were found, which can be explained by the low content of graphene oxide nanoplates in the MNP/GO composite (the presence of graphene and iron in the composites can be seen in the Raman spectra in Figure S5). It is also interesting that the intensity ratios of the PHB reflexes change after the introduction of different fillers into the scaffold (Figure 6b). The composite scaffolds show a predominance of the peak referenced to the (020) plane of the orthorhombic α-phase PHB, indicating anisotropic crystal growth in the b direction [68]. These data can be explained by the different orientations of PHB crystal lamellae along the fibers [69]. Table 4 summarizes the values of the calculated PHB crystallite sizes in the (020) and (110) planes.

As we have shown earlier using piezoresponse force microscopy, the higher the percentage of crystallinity of the PHB-based material, the stronger its piezoelectric properties [19]. In this respect, the properties of composite materials containing magnetically active nanoparticles were similar. The combination of crystalline and amorphous phases, a chaotic arrangement of individual crystallites in the structure, makes the surface of composite materials inhomogeneous in terms of the sign and magnitude of polarization. The occurrence of the surface microelectric field is significantly reflected in the value of the free surface energy, increasing the wettability of the material [70,71]. As mentioned above, this may be associated with a decrease in the degree of bacterial adhesion. However, a decrease in the number of bacteria on the surface of the material may not only indicate its anti-adhesive properties but also its bactericidal properties. Reviews by Li et al. and Mehrjou et al. list several piezoelectric materials for which a bactericidal effect has been shown [18,47]. There are a number of theories explaining the mechanisms of the microelectric field generated by the PEE on the surface of the piezoelectric material on bacterial cells. Among them are the generation of reactive oxygen species (ROS) and the disruption of the bioelectrical balance of the bacterial cell due to changes in its membrane potential under the influence of the electric field on the surface of the polymeric material.

## 3. Materials and Methods

### 3.1. Materials

The components of culture media (MgSO_4_·7H_2_O, FeSO_4_·7H_2_O, Na_2_MoO_4_·2H_2_O, sodium citrate, CaCl_2_, K_2_HPO_4_, KH_2_PO_4_ and sucrose), LB-bouillon in Miller’s modification, MRS-bouillon, isopropanol, chloroform, ethanol, potassium permanganate (KMnO_4_), sulfuric acid (H_2_SO_4_, 95%), hydrogen peroxide (H_2_O_2_, 30%), Iron(III) chloride hexahydrate (FeCl_3_·6H_2_O), iron(II) sulfate heptahydrate (FeSO_4_·7H_2_O), ammonium hydroxide (NH_4_OH) and citric acid (C_6_H_8_O_7_) were purchased from Merck (former Sigma-Aldrich, Darmstadt, Germany). Crystal violet was purchased from Acros Organics (Mumbai, India). Dry graphite was acquired from trading house Graphite Service LLC (St. Petersburg, Russia).

### 3.2. Synthesis of Magnetite Nanoparticles and Their Composites with Graphene Oxide

In our work, we used magnetite (iron oxide IV) nanoparticles surrounded by a shell of citric acid molecules by means of covalent modification preventing their adhesion (Figure 4e) as a magnetically active phase providing electromechanical coupling with PHB under the influence of a LFMF. Fe_3_O_4_ nanoparticles were synthesized by co-precipitation followed by covalent coating with citric acid to prevent aggregation as follows. Ferric (III) chloride hexahydrate (3.32 g) and ferrous (II) sulfate heptahydrate (1.22 g) were placed in a three-necked flask and set on a magnetic stirrer connected to the Schlenk system. The dry salts were subjected to vacuum degassing and then saturated with argon gas three times. Subsequently, 200 mL of deionized water was added to the flask, and the mixture was heated on the magnetic stirrer to 80 °C while being stirred at 1200 rpm. At this temperature, 20 mL of NH_4_OH was gradually added with a syringe. The heating process continued for 5 min. Next, 1.4 mL of 50% (*w*/*v*) citric acid was added to the solution, and the temperature was raised to 90 °C with continuous stirring for 90 min. Following this, the solution was decanted, and the resulting powders were washed with deionized water. The washing step was repeated until the pH of the solution reached neutrality. The powders were then precipitated using an external MF and subsequently freeze-dried (FreeZone 1 Liter Benchtop Freeze Dry System (Labconco, Kansas City, KS, USA)) for 2 days at −50 °C. Consequently, black magnetite powders were obtained. According to AFM data, the size of magnetite crystallites in them was 11.6 nm [72]. A SEM image of MNP is presented in Figure S2a. The data of iron and other element content in MNP obtained by the EDX method are presented in Figure S2d.

Partially reduced graphene oxide was synthesized through an enhanced Hummers method to attain a rich presence of oxygen functional groups on the surface. In a standard synthesis protocol, a mixture of concentrated H_2_SO_4_/H_3_PO_4_ (360:40 mL) in a ratio of 9:1 was introduced to a blend of 3.0 g of graphite flakes and 18.0 g of KMnO_4_. The resultant suspension was subjected to cooling at 0 °C within an ice bath for a duration of 2 h under continuous stirring. Following this, the reaction mixture underwent a heating process while being stirred at 50 °C for a duration of 12 h. Upon cooling to room temperature, the reaction mixture was poured onto 400 mL of ice supplemented with 3 mL of 30% H_2_O_2_. Subsequently, the reaction mixture underwent an overnight precipitation process and was subsequently decanted. The resultant product, presenting a brownish hue, was subjected to centrifugation (4500 rpm, 15 min) and subsequently washed with deionized water and 3% HCl in order to attain a neutral pH level. Then, the obtained GO was freeze-dried (using the FreeZone 1 Liter Benchtop Freeze Dry System) for 2 days at −50 °C. A SEM image of GO is presented in Figure S2b. The data of element content in GO obtained by the EDX method are presented in Figure S2e.

The direct contact between magnetite nanoparticles and GO provides the most complete electromechanical coupling of the filler phase and PHB, so we introduced these components into the polymer material in the form of a MNP-GO composite (Figure 4f). To synthesize the MNP-GO complex, the lyophilized partially reduced graphene oxide (0.1 g) was dispersed in a stirred round-bottom flask containing 100 mL of deionized water, followed by a 1 h sonication process (25 W, 40 kHz). FeSO_4_∙7H_2_O (0.61 g) and FeCl_3_∙6H_2_O (1.66 g) were dissolved in 75 mL of deionized water to prepare a ferrous solution. This ferrous solution was then introduced into the GO suspension. Subsequently, the suspension was subjected to argon bubbling to eliminate dissolved oxygen and ensure a protective environment during the reaction. The suspension was promptly immersed in a heated silicone bath, and incubation was conducted at 80 °C with vigorous stirring at 1200 rpm, along with the addition of 10 mL of 30 wt.% aqueous ammonia. This heating and stirring process was maintained for 5 min. Following this, 0.5 mL of 50% (*w*/*v*) citric acid was introduced into the solution, and the temperature was elevated to 90 °C, with continuous stirring for 90 min. Afterward, the solution was decanted, and the resultant powders underwent a series of washes with deionized water. The washing procedure was iterated until the solution’s pH reached neutrality. The resulting composite materials were collected using a magnet, washed with deionized water, and subjected to freeze-drying (employing the FreeZone 1 Liter Benchtop Freeze Dry System) for 2 days at –50 °C. The average atomic ratio of elements in the composite obtained by the EDX method was Fe 32–45%, C 20–30% and O 30–37% (Figure S2f*).* A SEM image of MNP/GO is presented in Figure S2c.

### 3.3. PHB Biosynthesis

*Azotobacter chroococcum* strain 7B was used for PHB biosynthesis. The bacteria were cultured in liquid Burke’s medium of the following composition: 1.6 mM magnesium sulfate; 0.18 mM iron (II) sulfate; 0.025 mM sodium molybdate; 1.5 mM sodium citrate; 0.9 mM calcium chloride; 4.6 mM dipotassium phosphate; 1.47 mM monobasic potassium phosphate; 88 mM sucrose; pH 7.3 ± 0.1 at 25 °C.

*A. chroococcum* was cultured for 72 h at 30 °C on an New Brunswick Innova-44 microbial incubator shaker (Edison, Rosemead, CA, USA) with stirring at 250 rpm (stroke 2 in). The biomass was dried and PHB was extracted from it with chloroform. The polymer was then precipitated from the extract by adding isopropanol. By repeating the procedures of dissolution and precipitation of the polymer, a high degree of purification was achieved. A detailed description of the methodology was presented earlier [73]. The purified PHB had a weight average molecular weight of 340 kDa, which was determined by viscometry.

### 3.4. Manufacturing of Polymer Films

The films were produced by the precipitation of polymers from the solution with solvent evaporation. To obtain the films from pure PHB, its 5% (*w*/*v*) solution in chloroform was poured into glass Petri dishes (KhimLaborPribor, Moscow region, Klin, Russia) at the rate of 300 μL per cm^2^. Petri dishes were placed at the bottom of a 25 cm high glass container with walls, which was covered with a paper filter and glass. This design ensured slow and uniform evaporation of chloroform (72 h at room temperature), which is a necessary condition for the formation of films with an even surface.

For the fabrication of PHB-MNP and PHB-MNP/GO composite films, the nanoparticle suspension was placed in a glass bottle, chloroform was poured so that their concentration in the colloidal system was 2% (*w*/*v*) and the bottle was closed with a lapped lid for dispersion for 2 h in an ultrasonic bath Elmasonic S10H (37 kHz, 30 W) (Elma, Pforzheim, Germany). The spatial orientation of MNP, both free and in the composite with GO, in the solution was random. This further determined their random spatial orientation in the polymer material. A 6.25% (*w/v)* solution of PHB in chloroform at a ratio of 1 to 4 by volume was added to the resulting dispersion and stirred for 2 h on a magnetic stirrer MSH-300 (Biosan, Riga, Latvia) using a glass-encased magnetic anchor. The composite solution thus obtained was poured into Petri dishes (KhimLaborPribor, Moscow region, Klin, Russia) as described above. The finished films had a thickness of 0.1 mm ± 0.03 (*n* = 54), and the weight ratio of magnetic nanoparticles in the composite material was 8% of the PHB weight [73]. 

### 3.5. Fabrication of Polymer Scaffolds by Electrospinning

Scaffolds were prepared by electrospinning on a self-made electrospinning machine (TPU, Tomsk, Russia). A solution of pure PHB in hexafluoroisopropanol with a final concentration of 3% (*w*/*w*) was used, or with the addition of composites whose weight was 8% of the initial weight. When nanoparticles were added, the solution was pre-dispersed in an ultrasonic bath, as described above. Electrospinning was carried out at 22 kV, 0.2 mA, a flow rate of the polymer solution of 0.28 mL/h and a rotation speed of the cylindrical manifold (*Ø* = 2.5 cm) of 200 rpm. The finished scaffolds had a thickness of 0.076 mm ± 0.01 (*n* = 36). The weight of 1 cm^2^ was 1.6 mg ± 0.2 (*n* = 36).

### 3.6. Study of Bacterial Adhesion and the Effect of NMP on the Density of Suspension Cultures

We selected bacteria for modeling their interaction with the studied materials according to the following criteria: these microorganisms should be facultative anaerobes, non-spore-forming and non-motile, and at the same time, should differ greatly in cell wall structure. According to the above conditions, two bacterial species were selected: Gram-positive *Lactobacillus fermentum* strain 90 TS-4, which is not among the known motile *Lactobacillus* strains [74], and Gram-negative *Escherichia coli* strain BL21, which does not express flagellar genes and has lost motility [75]. For *E. coli* BL21, it was previously shown to have adhesive activity to abiotic surfaces [76].

The obtained film and scaffold samples from pure PHB and from the PHB-MNP and PHB-MNP/GO composites, 10 by 10 mm in size, were placed in 2-milliliter Eppendorf-type tubes with an inner diameter of 9 mm, which ensured that the polymer samples were fixed in them. They were sterilized by autoclaving for 15 min at 112 °C.

*E. coli* and *L. fermentum* were cultured in liquid media. *E. coli* was cultured in Luria-Bertani broth in Miller’s modification of the following composition (weight/volume percentage concentration is given hereafter): 0.5% yeast extract; 1% tryptone; 0.17 M NaCl; pH 7.1 ± 0.1 at 25 °C. *L. fermentum* was cultured in De Man, Rogosa and Sharp broth: 1% casein peptone; 0.8% meat extract; 0.4% yeast extract; 0.1% Tween-80; 0.1 M D(+) glucose; 11.5 mM K_2_HPO_4_; 8.8 mM diammonium citrate; 61 mM sodium acetate; 1.7 mM magnesium sulfate; 0.26 mM manganese sulfate; pH 5.7 ± 0.2 at 25 °C. The density of suspensions was determined by the turbidimetric method on a SF-2000 spectrophotometer (OKB-Spectr, Sankt-Peterburg, Russia) at a wavelength of *λ* = 600 nm and an optical path length of 1 cm. For correct analysis of the samples with OD values exceeding 0.8 units, they were diluted with K-phosphate buffer for the required number of times.

Bacteria were cultured on a New Brunswick Scientific classic series microbiological shaker–incubator (Edison, Rosemead, CA, USA) with a stroke of 0.75 inches at 37 °C and stirred (*E. coli* at 280 rpm; *L. fermentum* at 120 rpm) for 4–6 h until the bacterial suspensions reached OD values of 0.8–1. The suspensions were then diluted with culture medium to an OD of 0.01, added in 1.5 mL amounts into Eppendorf-type tubes, which could be empty or contain sterile samples of films or scaffolds, and hermetically sealed with lids. The tubes were placed in a thermostat maintaining 37 °C for 24 h (*E. coli*) or 16 h (*L. fermentum*). At the same time, some samples were exposed to a LFMF (*B_m_* = 68 mT, *f* = 0.67 Hz) in a self-made MF setting located in the same thermostat.

After the end of the incubation, the polymer samples were removed, and the OD of the remaining bacterial suspensions was measured. The degree of bacterial adhesion to the polymeric samples was determined according to the modified methods of Genevaux and Pratt and Kolter [77,78]. Polymer samples were gently flushed with distilled water to remove weakly bound cells, incubated for 30 min at 80 °C in a Thermomixer comfort (Eppendorf, Hamburg, Germany) to dry and fix bound cells, and then stained with 0.1% (*w*/*v*) aqueous solution of crystal violet for 5 min at room temperature. The stained samples were gently washed with distilled water and then placed in 96% ethanol for 60 min at room temperature for crystal violet extraction. The OD of the extracts obtained was determined at a wavelength of 565 nm and an optical path length of 1 cm. If necessary, samples with a high OD value were diluted with 96% ethanol.

The OD_(Sample)_ value, which reflected the number of bound bacteria, was found by considering the background binding of crystal violet to polymer samples. To assess the background binding of the dye to the polymer samples, they were incubated in culture medium without bacteria and then stained, similar to the experimental samples.

### 3.7. Microscopy

SEM was performed on a TM3000 tabletop microscope (Hitachi, Tokyo, Japan) and a JSM-6380LA Analitical scanning electron microscope (JEOL, Tokyo, Japan) with a spot size of 30 at 15 kV and 20 kV, respectively. The Pt/Pd layer, 15 nm thick, was sputtered on an B*3 Ion coater (Eiko, Tokyo, Japan).

AFM was performed on an Integra Prima SI microscope (NT-MDT, Zelenograd, Russia). Silicon cantilevers NSG01 (TipsNano, Moscow, Russia) with a typical force constant of 1.45–15.1 N/m and a tip curvature radius of 10 nm were used. The images were recorded in semi-contact mode with the air, with a scanning resonance frequency of 87–230 kHz. Topography and phase signals were captured during each scan. The images were captured with 512 × 512 points. Image processing was carried out using Image Analysis 3.5 (NT-MDT, Moscow, Russia, https://www.ntmdt-si.com/resources/webinars/image-processing-and-analysis-in-scanning-probe-microscopy, 8 May 2019) and FemtoScan Online 2.4.10 (Advanced Technologies Center, Moscow, Russia, http://www.nanoscopy.net/en/femtoscan.php?t=7, 4 July 2019) software. To describe the surface of polymer film samples, a roughness analysis of their profile was performed using 10 AFM scans according to the ISO 25178 standard [79]. The arithmetical mean height of the scale-limited surface (*S_a_*, [nm]) was calculated from 5 × 5 μm^2^ scans as the arithmetic mean of the absolute value of the ordinate in the area of definition (A):

(1) $$S_{a}=\frac{1}{A}\iint_{A}\left|z(x,y)\right|dxdy$$

The scale-limited surface boundary area ratio of the scale-limited surface (*Sdr*, [%]) was calculated from scans of 50 × 50 μm^2^ as the percentage of the definition area’s additional surface area contributed by the texture as compared to the planar definition area:

(2) $$S_{dr}=\frac{1}{A}\left[\iint_{A}\left(\sqrt{\left[1+{\left(\frac{𝜕z(x,y)}{𝜕x}\right)}^{2}+{\left(\frac{𝜕z(x,y)}{𝜕y}\right)}^{2}\right]}-1\right)dxdy\right]$$

### 3.8. Energy Dispersive X-ray Spectroscopy (EDX)

A Quanta 600 electron microscope (Thermo Fisher, Waltham, MA, USA) equipped with an energy dispersive spectroscopic analyzer (EDRA) using a SEM–EDX–EMAX analysis instrument (Thermo Fisher, Waltham, MA, USA) at an accelerating voltage of 10 kV was used to determine the chemical composition of the samples.

### 3.9. X-ray Structural Analysis (XRD)

The ultra-structure of the materials was analyzed on a diffractometer XRD-6000 (Shimadzu, Kyoto, Japan) with X-ray radiation CuKα in the range of scattering angles from 10 to 40°. The wavelength of X-ray radiation was 0.15406 nm. The size of PHB crystallites in the planes (020) and (110) was calculated according to the formula of Scherrer [80]:

(3) $$D=\frac{K\lambda }{\beta coscos\;\theta \;}$$

where *λ* [nm] is the X-ray wavelength; *β* [rad] is the peak width at half-height; *θ* [rad] is the diffraction angle of scattering; *K* is a dimensionless particle shape factor equal to 0.9.

### 3.10. Fourier-Transform Infrared (FTIR) Spectroscopy

Spectra were collected with the use of a Spectrum Two FT-IR Spectrometer (PerkinElmer, Waltham, MA, USA) equipped with a room-temperature LiTaO_3_ (lithium tantalate) MIR detector with a SNR of 9300:1 and an optical system with KBr windows for data collection over a spectral range of 550–2000 cm^−1^ at a best resolution of 0.5 cm^−1^.

### 3.11. Contact Angle

The water wetting contact angle was determined by the drop deposition method using distilled water as a wetting liquid at a room temperature of 22 °C and a humidity of 40%. The volume of the drop was 5 μL. Eight measurements were taken for each sample.

The water contact angle of bacteria was determined according to the method of Busscher et al. with minor modifications [81]. The bacterial suspension culture was precipitated by centrifugation at 2000× *g* and was washed twice from the residual culture medium by resuspension in distilled water and re-precipitation. The bacteria were then concentrated on a nitrocellulose membrane filter with a pore size of 0.45 μm. Filters with a uniform bacterial layer were dried to a constant mass value and then glued onto a slide.

When the contact radius of a droplet with a solid surface is the same, the actual contact area with a rough surface will be greater than the contact area with a smooth surface. The ratio of these areas is called the roughness factor (r) [82]:

(4) $$r=\mathrm{roughness}\;\mathrm{factor}=\frac{actual\;surface}{geometric\;surface}$$

The same parameter, expressed as a percentage of the corresponding areas, is commonly referred to as the “Surface area ratio” (Sdr). Having determined the values of *r* and *θ*, it is possible to calculate the value of the contact angle θ* on a perfectly smooth surface by applying the Wenzel ratio [56,82]:

*r* = cos *θ*/cos θ* (5)

### 3.12. Differential Scanning Calorimetry

The thermal properties of films and scaffolds made of pure PHB and composites were measured by DSC on a DSC 204 F1 Phoenix (Netzsch, Selb, Germany). Samples weighing 1–4 mg were sealed in a 25 μL aluminum crucible and heated from 25 to 200 °C at a rate of 10°/min in a nitrogen atmosphere. The onset and peak temperatures of heat capacity change were designated as T^onset^ and T^peak^ melting points, respectively. The temperature determination error did not exceed 1 °C, and the enthalpy of phase transition (for the melting enthalpy) was 2 J/g.

The crystallinity of the PHB component (*X_c_*, [%]) was calculated based on the following expressions [83]:

- For pure PHB:
  (6) $$X_{C}=\frac{{∆H}_{m}}{{∆H}_{0m(PHB)}}$$
- For composites of PHB with magnetic nanoparticles:
  (7) $$X_{C}=\frac{{∆H}_{m}}{{∆H}_{0m(PHB)}\times \omega_{\left(PHB\right)}}$$
  where the value ∆*H*_0*m(PHB)*_ is the theoretical value of the thermodynamic enthalpy of melting that would be obtained for a 100% crystalline sample of PHB (146.6 J/g); *H_m(PHB)_* is the apparent enthalpy of melting corresponding to the PHB component; and *ω_(PHB)_* is the weight fraction of PHB in the composite. The data are presented as the average of three measurements.

### 3.13. Statistical Analysis

Statistical analysis was performed using the OriginPro software v9.2.214 (OriginLab Corporation, Northampton, MA, USA, https://store.originlab.com/store/index.aspx?CID=52, 29 November 2019). The Mann–Whitney U-criterion with a significance level of *p* < 0.05 was used to test the reliability of differences between pairs of compared data series. Data were averaged with the standard error to the mean (±SD). A *p*-value less than 0.05 was considered statistically significant. 

## 4. Conclusions

During the development of bioengineered materials for medical applications, the modification of material composition, such as the addition of magnetically active particles to PHB in the case of our experiment, leads to a complex change in their physicochemical properties. Additional effects on electroactive materials, in order to induce the piezo effect, are also aimed at changing the properties of their surface. These changes should be reflected in the interaction of cells, including bacterial cells, with their surface. Using *E. coli* and *L. fermentum,* we investigated how the combination of different physicochemical properties of PHB-based materials affects the adhesion of these model microorganisms. Since different species and even strains of bacteria differ in their adhesive properties, changes in the properties of the polymer material can be reflected differently in their binding, which is, on the one hand, a prerequisite for the development of bioengineered materials with a targeted action against specific groups of bacteria, and, on the other hand, allows us to select a model for indicating changes in surface properties.

The addition of MNP and MNP/GO to the polymeric material, the mass of which amounted to 8% of the PHB mass, resulted in significant changes in its morphology and physicochemical properties: the crystallinity of the material, its hydrophobicity and charge, as well as surface roughness. Nanoparticles differ greatly in their physicochemical properties from those of the polymer, changing the structural organization of its chains: the ratio of crystalline and amorphous phases and their mutual location, which is reflected in the roughness of its surface. The degree of crystallinity of composite films and scaffolds according to DSC data was on average 23% lower than that of pure PHB samples. At the same time, the crystallite size calculated from XRD analysis data increased on average by 1.4 and 2.3 times for films, and by 1.7 and 1.3 times for scaffolds in the (020) and (110) planes, respectively. Comparison of micro- and nanotopography of polymer films showed that the value of *S_a_*, characterizing the average surface roughness, for films made of pure PHB was two to three times lower than for films made of composites.

The change in the crystallinity of PHB-based composites compared to pure polymers, as well as the exposure of polar and ionizable groups of microparticles to the surface of the composite material, can change its surface energy and affect the surface charge value of the polymer material. Under the conditions of our experiment, this effect was relatively weakly expressed. Considering the differences in film roughness, the values of the contact angle of the composites were lower by 2.4% for PHB-MNP and by 0.6% for PHB-MNP/GO films. From the staining of the polymer films with crystal violet, we showed that the addition of nanoparticles in its composition imparted a negative charge to the surface. The effect was more pronounced (1.5-fold) for PHB-MNP than for PHB-MNP/GO films.

The change in the physicochemical properties of the polymer material affected the adhesion of model microorganisms to the surface of the films and scaffolds, and this effect was selective. The adhesion of *L. fermentum* to both composite materials was similar and increased by 1.8 and 1.4 times for films and scaffolds, respectively, while the adhesion of *E. coli* increased only to the PHB-MNP/GO samples (by 4.4 and 2.8 times for films and scaffolds, respectively) and did not differ to samples made of pure PHB and PHB-MNP composite. The results of our experiments reflect the combined effect of all of the factors that we observed: surface topography, its hydrophobicity and charge. Moreover, the Gram-positive and Gram-negative model microorganisms used by us also differ in the structure of the cell wall and their hydrophobicity and accordingly, have different sensitivity to each independently of the factors changed in the experimental system. As a result, the resulting factors enhancing and weakening the binding to the substratum of *E. coli* and *L. fermentum* were multidirectional.

Exposure to a LFMF had a multidirectional effect on the adhesion of model microorganisms. It did not affect the binding of *L. fermentum* to pure PHB and PHB-MNP/GO films and scaffolds but decreased the adhesion to PHB-MNP samples by 1.7 times. MNP were added to the composite material in order to induce the piezo effect of PHB under the influence of an alternating magnetic field through electromechanical coupling. In our experiments, the degree of bacterial adhesion in the model with *L. fermentum* served as an indicator of the manifestation of the piezo effect on the surface of the PHB-MNP composite and of the absence of evidence of its expression, contrary to our expectations, on the surface of the second PHB-MNP/GO composite.

In our work, the LFMF had an effect on *E. coli* adhesion to pure PHB films and scaffolds. The design of our experiment does not allow us to speculate on the mechanisms underlying this phenomenon, which is still poorly understood, but these data will add to the general pool of information in the field of magnetobiology, and we believe that they will help us to find an answer in the future. The adhesion of *E. coli* to PHB-MNP samples under the action of the LFMF did not change, and increased by 2.9 and 2.3 times for films and scaffolds from PHB-MNP/GO, respectively. The lack of effect on the PHB-MNP samples was probably due to the manifestation of the anti-adhesive action of the PEE.

The current literature supports the idea that the view of the role of microbiota in tissue regeneration should not be limited to its consideration as a cause of the infectious process but should be extended to the study of its therapeutic value. In light of this, new bioengineering approaches and materials that allow for selective action on the bacterial community open up prospects for their application as potential tools for regenerative medicine.

## Figures and Tables

**Table 1:** Average roughness profiles of AFM scans of polymer films (5 µm × 5 µm scan size) from PHB, PHB-NMP and PHB-MNP/GO.

Sample	*R_a_*, nm
PHB, upper side	68 ± 6.3
PHB, lower side	18 ± 9.9
PHB-MNP, upper side	84 ± 25.4
PHB-MNP, lower side	54 ± 8.3
PHB-MNP/GO, upper side	154 ± 36.7
PHB-MNP/GO, lower side	59 ± 6.5

**Table 2:** Contact angles (*θ*) and surface area ratio (*Sdr*).

Sample	*θ* [°]	*Sdr* [%]	θ* [°]
PHB, upper side	72.8 ± 1.7	8.19 ± 1.8	71.2
PHB, lower side	71.4 ± 0.8	1.20 ± 0.3	71.2
PHB-MNP, upper side	72.1 ± 0.9	11.76 ± 0.3	69.6
PHB-MNP, lower side	71.7 ± 1.6	10.94 ± 1.1	69.4
PHB-MNP/GO, upper side	74.6 ± 2.8	19.42 ± 3.0	70.8
PHB-MNP/GO, lower side	72.8 ± 1.2	10.47 ± 0.9	70.7
*E. coli* BL21	50.3 ± 1.9		
*L. fermentum* 90T-C4	40.2 ± 3.9		

θ* corrected contact angles calculated from relation (5).

**Table 3:** Thermophysical properties of films and scaffolds from PHB, PHB-MNP and PHB-MNP/GO.

Samples	ΔH_m_ [J/g]	T_m_ [°C]	X_c_ [%]
PHB film	110.4	179.3	75.3 ^P^
PHB-MNP film	87.4	179.0	54.8 ^C^
PHB-MNP/GO film	92.6	178.1	58.1 ^C^
PHB scaffold	93.6	176.2	63.8 ^P^
PHB-MNP scaffold	80.8	175.4	50.7 ^C^
PHB-MNP/GO scaffold	78.9	175.9	49.5 ^C^

X_c_ ^P^—crystallinity of pure PHB was calculated by the relation (6); X_c_ ^C^—crystallinity of composite polymer materials was calculated by the relation (7).

**Table 4:** PHB crystallite size in the (020) and (110) planes [nm].

Samples	(020)	(110)
PHB film	16.2	5.2
PHB-MNP film	21.3	12.1
PHB-MNP/GO film	22.2	11.6
PHB scaffold	6.2	13.0
PHB-MNP scaffold	11.7	15.7
PHB-MNP/GO scaffold	8.9	16.5

The crystallite size of PHB was calculated from relation (3).

## Data Availability

Data is contained within the article and Supplementary Materials.

## Supplementary Materials

The following supporting information can be downloaded at: https://www.mdpi.com/article/10.3390/ijms25010208/s1.
