# Supplementary material for: Adhesion of Escherichia coli and Lactobacillus fermentum to Films and Electrospun Fibrous Scaffolds from Composites of Poly(3-hydroxybutyrate) with Magnetic Nanoparticles in a Low-Frequency Magnetic Field
Source: Int J Mol Sci. 2023 Dec 22;25(1):208. doi: 10.3390/ijms25010208 (PMC10778586; doi:10.3390/ijms25010208)
Supplement: Supplementary file 1 [file ijms-25-00208-s001.zip › ijms-2723930-supplementary.pdf]

Supplementary materials

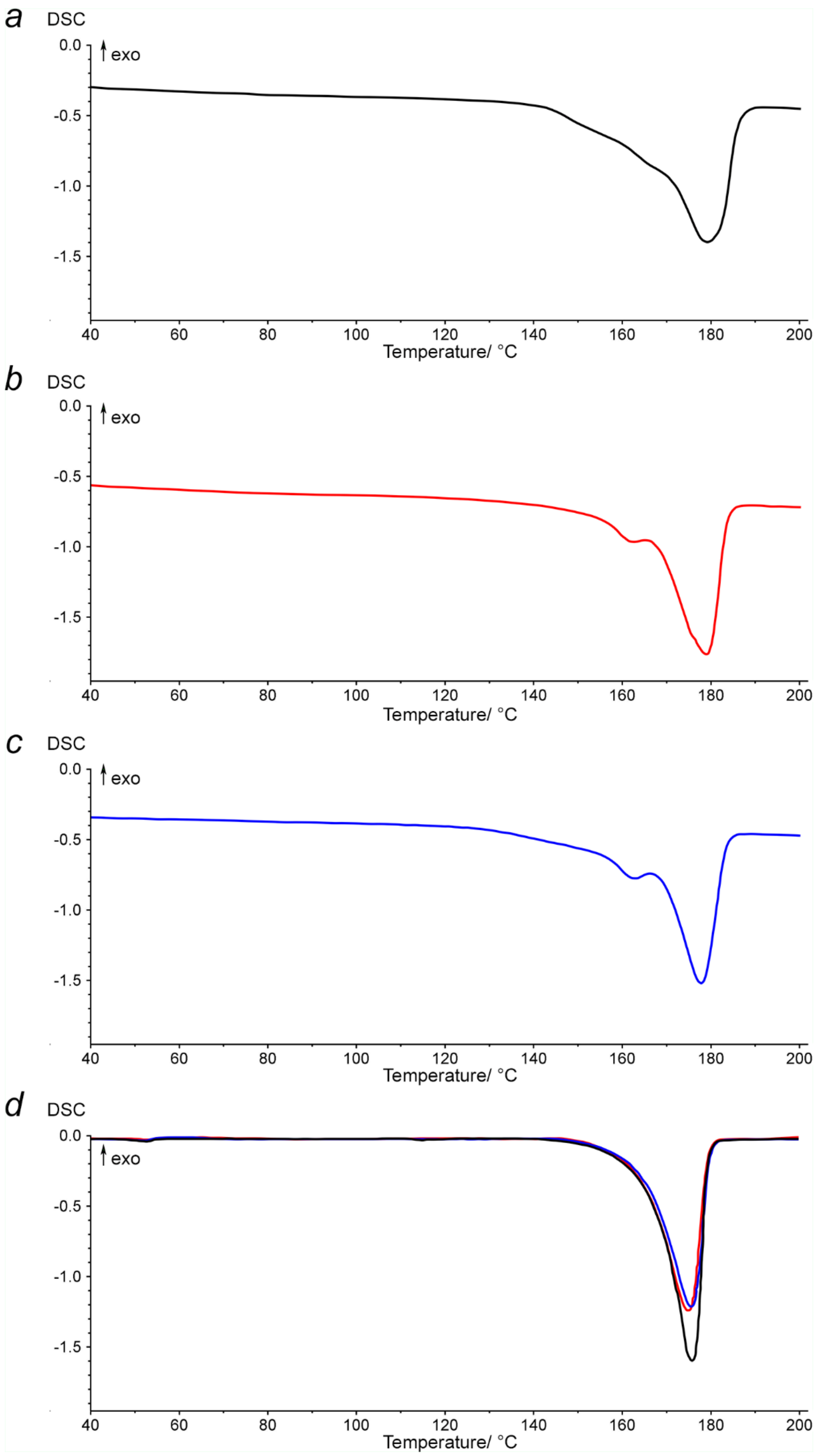

Figure S1. DSC measurements of the (***a***) film made of pure PHB; (***b***) film made of composite PHB-MNP; (***c***) film made of composite PHB-MNP/GO; (***d***) scaffolds made of pure PHB (black line), scaffolds made of composite PHB-MNP (red line) and scaffolds made of composite PHB-MNP/GO (blue line).

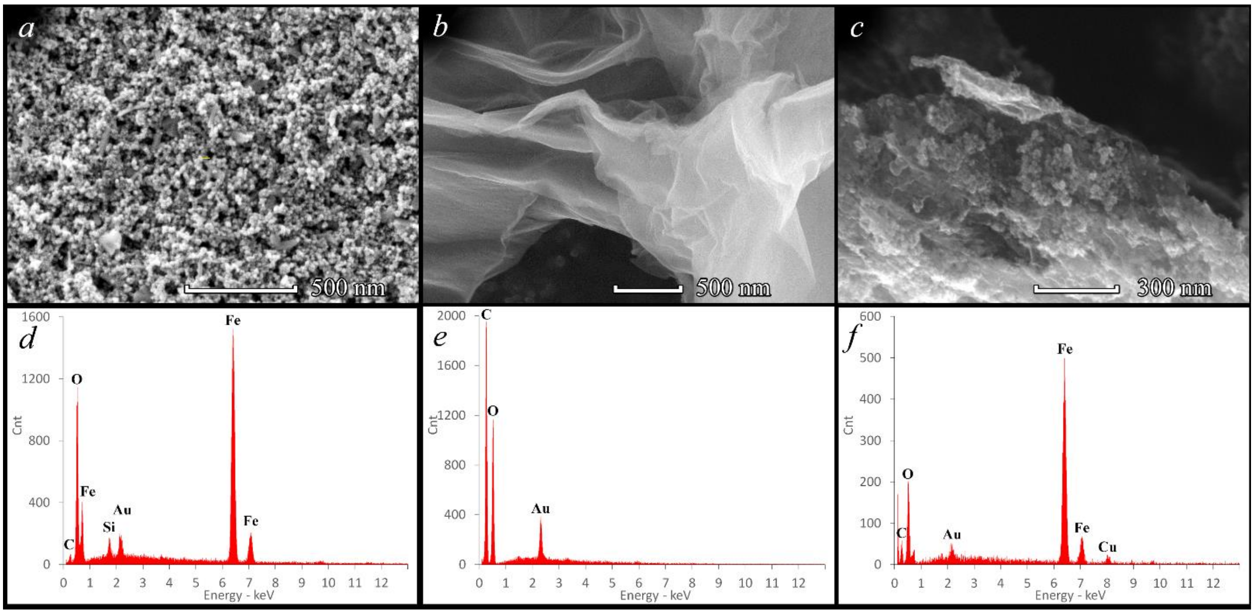

Figure S2. SEM images for MNP, GO and composite MNP/GO (***a*** – ***c***)**.** Energy dispersive X-ray spectrum of MNP (*d*), GO (*e*) and MNP/GO (*f*).

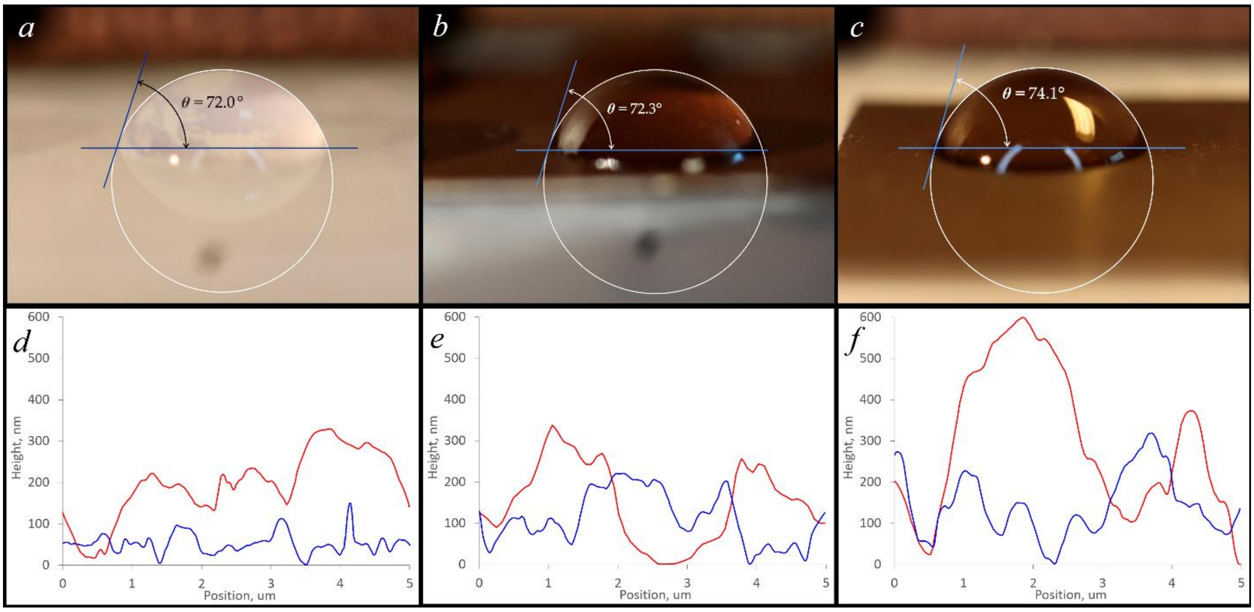

Figure S3. Water contact angle profiles of upper side of PHB (*a*), PHB-MNP (*b*) and PHB-MNP/GO (*c*) polymer films. AFM scans of roughness profiles of upper (red line) and lower (blue line) sides of PHB (*d*), PHB-NMP (*e*) and PHB-MNP/GO (*f*) polymer films.

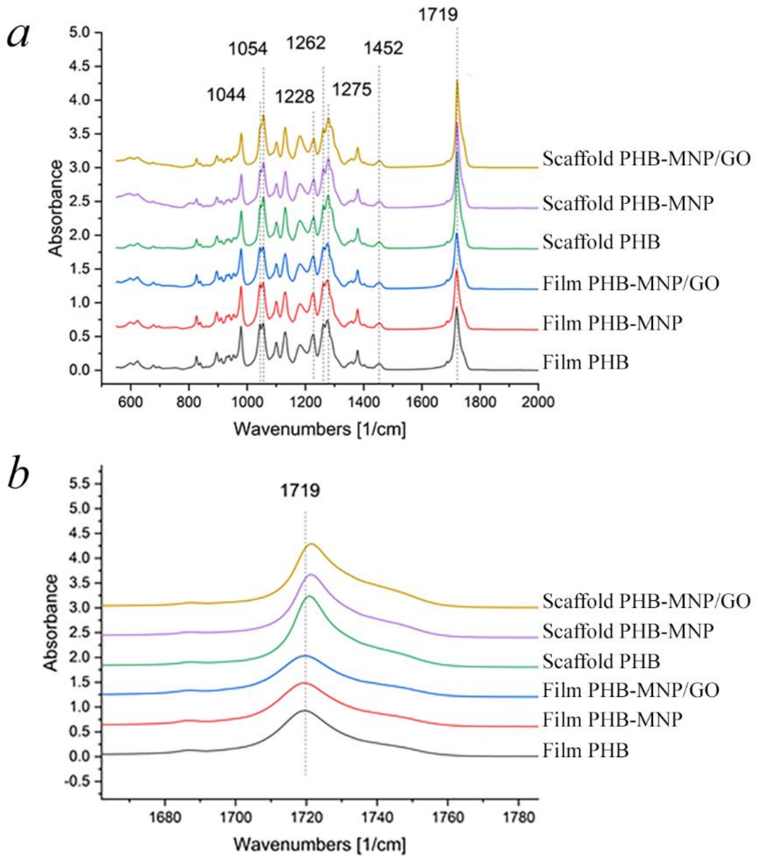

Figure S4. FTIR spectra of films and scaffolds of pure PHB and its composites with MNP and MNP/GO. The base line is subtracted. The spectra are normalized to the intensity of the 1452 band (asymmetric deformation vibrations of hydrogens in the CH_3 groups). Spectra were collected between 400 and 2000 cm^{-1} (*a*) and at 1660 and 1790 cm^{-1} (*b*).

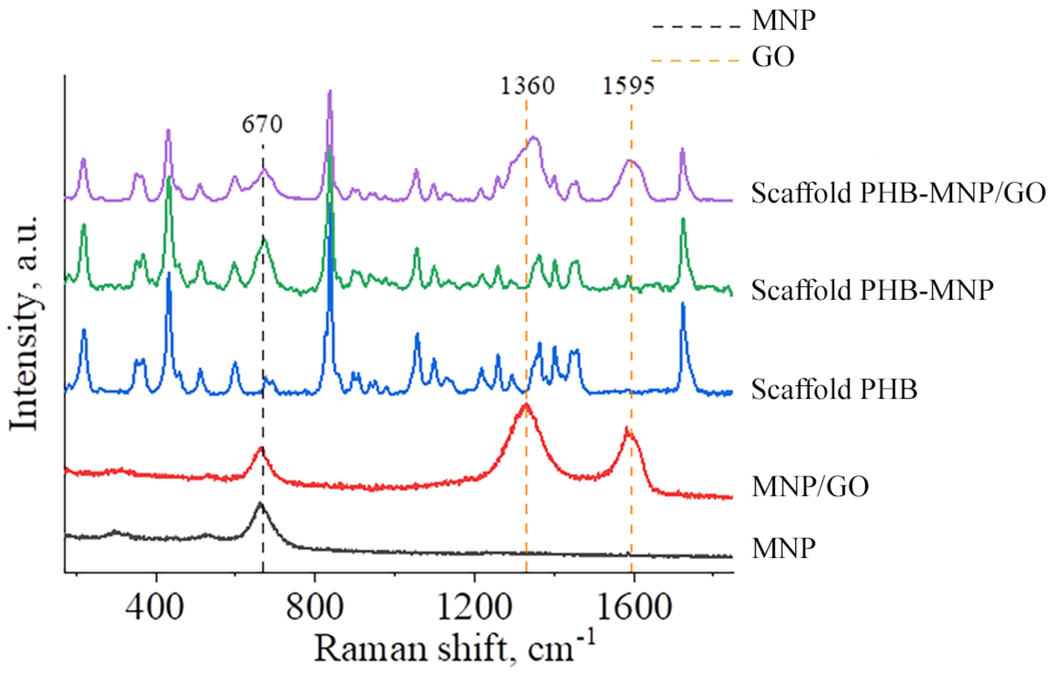

Figure S5. Raman spectra of the MNP, MNP/GO, pure PHB and composite scaffolds.
